# Evolution of context dependent regulation by expansion of feast/famine regulatory proteins

**DOI:** 10.1186/s12918-014-0122-2

**Published:** 2014-11-14

**Authors:** Christopher L Plaisier, Fang-Yin Lo, Justin Ashworth, Aaron N Brooks, Karlyn D Beer, Amardeep Kaur, Min Pan, David J Reiss, Marc T Facciotti, Nitin S Baliga

**Affiliations:** Institute for Systems Biology, Seattle, WA USA; Molecular and Cellular Biology Program, University of Washington, Seattle, WA USA; Department of Biomedical Engineering, University of California, Davis, CA USA; Genome Center, University of California, Davis, CA USA; Department of Microbiology, University of Washington, Seattle, WA USA; Department of Biology, University of Washington, Seattle, WA USA

**Keywords:** Transcription factor, Expansion, Systems biology

## Abstract

**Background:**

Expansion of transcription factors is believed to have played a crucial role in evolution of all organisms by enabling them to deal with dynamic environments and colonize new environments. We investigated how the expansion of the Feast/Famine Regulatory Protein (FFRP) or Lrp-like proteins into an eight-member family in *Halobacterium salinarum* NRC-1 has aided in niche-adaptation of this archaeon to a complex and dynamically changing hypersaline environment.

**Results:**

We mapped genome-wide binding locations for all eight FFRPs, investigated their preference for binding different effector molecules, and identified the contexts in which they act by analyzing transcriptional responses across 35 growth conditions that mimic different environmental and nutritional conditions this organism is likely to encounter in the wild. Integrative analysis of these data constructed an FFRP regulatory network with conditionally active states that reveal how interrelated variations in DNA-binding domains, effector-molecule preferences, and binding sites in target gene promoters have tuned the functions of each FFRP to the environments in which they act. We demonstrate how conditional regulation of similar genes by two FFRPs, AsnC (an activator) and VNG1237C (a repressor), have striking environment-specific fitness consequences for oxidative stress management and growth, respectively.

**Conclusions:**

This study provides a systems perspective into the evolutionary process by which gene duplication within a transcription factor family contributes to environment-specific adaptation of an organism.

**Electronic supplementary material:**

The online version of this article (doi:10.1186/s12918-014-0122-2) contains supplementary material, which is available to authorized users.

## Background

Expansion of transcription factor (TF) families via gene duplication enables an organism to adapt to new environments by providing a means to rewire its gene regulatory network [1]. The process of rewiring is accomplished through natural selection of random mutations that maneuver each TF homolog into a distinct niche. Mutations that alter the set of target genes regulated by a TF can lead to functionally different effects. This process of functional divergence has two primary outcomes: 1) neo-functionalization where a TF gains a new function not present in the ancestral TF, and 2) sub-functionalization where the homologous TFs divide the functions of the ancestral TF [1,2]. Mutations that change the context where TF homologs are expressed can also be very important as they can relocate an advantageous function to a new context [2]. This complementary process of contextual divergence also has two primary outcomes: 1) neo-contextualization can bring an advantageous function to a new context, and 2) sub-contextualization where TF homologs split up the contexts of the ancestral TF [2,3]. Thus, mutations causing functional and contextual divergence of duplicated TFs allow organisms to explore a large space of new environmental or nutritional niches. Interestingly, homologs that are co-expressed in a particular context tend to have divergent DNA recognition motifs (i.e., they act in similar contexts but regulate different genes) and homologs expressed in different contexts often retain similar DNA recognition motifs (i.e., they regulate the same genes albeit in different environments) [2]. Thus, through TF duplication followed by functional and contextual divergence an organism can rewire its gene regulatory network to deal with new nutritional and environmental challenges.

Feast/famine regulatory proteins (FFRPs) [4] or Lrp-like proteins [5] of the Lrp/AsnC family (PF01037, AsnC_trans_reg) represent one of the oldest and largest families of prokaryotic transcriptional regulators. This ancient family of TFs is found both in archaea and bacteria suggesting that their common ancestor had at least one FFRP-like protein [6]. It is striking that on average each sequenced archaeal genome encodes 5 (±4) FFRPs, which suggests that expansions in the FFRP family had already occurred in a common ancestor (Additional file 1: Table S1). For instance, in the archaeal family of halobacteriaceae FFRP expansions have led to an average of 10 (±2) FFRP homologs per sequenced genome. Thus, it is safe to assume that the FFRP gene family has evolved through numerous expansions prior to and after evolution of the archaeal lineage [7] and that these expansions provide one possible means for organisms to adapt to changes in nutritional and/or environmental conditions [6,7].

Our research focuses on the genome of *H. salinarum* NRC-1 from the halobacteriaceae family, which encodes eight full-length FFRP homologs as well as an additional putative FFRP homolog that is missing a DNA binding domain (Additional file 2: Figure S1) [8]. Structurally FFRP proteins are comprised of a helix-turn-helix (HTH) DNA binding domain connected through a flexible linker to a “regulation of amino acid metabolism” (RAM) domain that typically binds amino acids to modulate regulatory activity [6,9-18]. RAM domains in some FFRPs have strong specificity for a single amino acid [14,17,18], some are activated by two or more amino acids [13,15,16], and others have evolved specificity to non-amino acid effector molecules [6,10,12]. The presence of a TrkA-C domain [19] in Trh2 and a TRASH domain [20,21] in VNG1179C suggest these FFRPs may be involved in the sensing and regulation of genes in response to changes in K+/NAD + and metals (e.g. Cu(II) [20]), respectively (Additional file 2: Figure S2). However, the contexts in which the eight FFRPs act and the specific genes they regulate are largely unknown. This information is essential to understand how the eight FFRP family members in *H. salinarum* NRC-1 have functionally and contextually diverged.

Here we have characterized the functional and contextual divergence of expanded FFRP family members in *H. salinarum NRC-1* to understand how TF homologs evolve to occupy different niches. The key features defining an FFRP’s niche are the repertoire of target genes that it regulates, the contexts in which it is expressed, and the effector molecules that modify its activity. We experimentally mapped genome-wide binding locations for all eight FFRPs, analyzed their expression across 466 gene expression microarrays from 35 different growth conditions which mimic environmental and nutritional contexts *H. salinarum NRC-1* is likely to experience in the wild, and inferred their effector-molecule preferences. This integrated analysis provided evidence for both functional and contextual divergence in the evolution of distinct conditionally active regulatory networks for five of the eight FFRPs. We have performed follow-up experiments that validate conditional regulation by two FFRPs, and demonstrate a context dependent fitness benefit for the regulation. Our results demonstrate that the eight FFRPs in *H. salinarum* NRC-1 have evolved to occupy distinct niches through variations in one or all of the three known determinants of their functions: which genes they regulate, when they are expressed, and what effector-molecules they bind. Importantly, these results illustrate how interrelated variations in these three properties tune function of each FFRP to the environmental context in which it acts.

## Results and discussion

### Evolution of Homologous FFRPs in *H. salinarum* NRC-1

Duplication events leading to eight full length homologous FFRPs in *H. salinarum* NRC-1 (AsnC (VNG1377G), Trh2 (VNG1285G), Trh3 (VNG1816G), Trh4 (VNG2094G), Trh6 (VNG1351G), Trh7 (VNG1123G), VNG1179C, and VNG1237C) occurred long before *H. salinarum* NRC-1 diverged from other phylogenetically related archaea. This assertion is supported by the fact that on average sequenced archaeal genomes have 5 ± 4 FFRP homologs suggesting that progenitors for many of the FFRPs in *H. salinarum* NRC-1 were likely present in a common ancestor of most archaeal lineages. Given this amount of time, it is likely that evolutionary processes would generate observable amounts of functional and contextual divergence between the homologous *H. salinarum* NRC-1 FFRPs. Additionally, the observation that the halobacteriaceae family, that includes *H. salinarum* NRC-1, has an average of 10 ± 2 FFRP homologs, which demonstrates that recent expansions of FFRPs have occurred within this family. An excellent example of recent expansions within halobacteriaceae and of functional divergence between FFRP homologs is the fusion of new functional domains TrkA-C and TRASH to Trh2 and VNG1179C, respectively. The fusion of TrkA-C is restricted to the halobacteriaceae (Additional file 3: Table S2) and fusion of the TRASH domain is restricted to the phyla crenarchaeota and euryarchaeota (Additional file 4: Table S3). We then hypothesized that less obvious functional divergence may be observed by analyzing mutations accrued in protein coding sequences. Functional divergence of gene family members at the protein level can be quantified as changes in conservation at specific residues between an FFRP and its related homologs compared to another FFRP and its related homologs (previously described by Gu, et al. 2013 [22]). We applied this approach to full length protein sequences to estimate the pairwise coefficient of functional divergence (type-I functional divergence or θ_I_) between all FFRPs. We found that each FFRP had significant evidence for functional divergence from every other FFRP (θ_ij_ >0 and *p*-value ≤0.05, Table 1). Through these evolutionary analyses, we have provided evidence that some of the FFRPs in *H. salinarum* NRC-1 are as old as the archaeal lineage and that there have been recent expansions within the halobacteriaceae family. We also provide evidence that each FFRP has significantly functionally diverged at the protein sequence level, and in subsequent sections we will explore the implications of this sequence level divergence on the function of each FFRP.

**Table 1:** **Pairwise functional divergence of FFRP family members**

	**Type-I functional divergence (θ**_**ij**_ **± SE)**							
***p*** **-values**	**AsnC**	**Trh2**	**Trh3**	**Trh4**	**Trh6**	**Trh7**	**VNG1179C**	**VNG1237C**
**AsnC**		0.47 ± 0.16	0.64 ± 0.11	0.33 ± 0.09	0.54 ± 0.13	0.51 ± 0.11	0.57 ± 0.19	0.42 ± 0.16
**Trh2**	3.3 × 10^−3^		0.60 ± 0.09	0.64 ± 0.09	0.44 ± 0.10	0.66 ± 0.09	0.48 ± 0.11	0.40 ± 0.11
**Trh3**	4.1 × 10^−9^	2.7 × 10^−11^		0.50 ± 0.06	0.58 ± 0.08	0.73 ± 0.07	0.57 ± 0.09	0.65 ± 0.08
**Trh4**	4.6 × 10^−4^	7.1 × 10^−13^	1.5 × 10^−15^		0.44 ± 0.07	0.72 ± 0.07	0.79 ± 0.08	0.44 ± 0.08
**Trh6**	3.2 × 10^−5^	3.3 × 10^−5^	2.8 × 10^−11^	1.4 × 10^−10^		0.83 ± 0.08	0.49 ± 0.12	0.60 ± 0.11
**Trh7**	4.4 × 10^−6^	2.2 × 10^−14^	2.6 × 10^−23^	1.4 × 10^−24^	4.1 × 10^−23^		0.87 ± 0.08	0.87 ± 0.08
**VNG1179C**	3.3 × 10^−3^	3.8 × 10^−5^	9.3 × 10^−11^	6.5 × 10^−24^	4.7 × 10^−5^	6.0 × 10^−24^		0.60 ± 0.10
**VNG1237C**	7.6 × 10^−3^	5.5 × 10^−4^	6.8 × 10^−14^	1.5 × 10^−8^	2.5 × 10^−7^	3.9 × 10^−24^	2.0 × 10^−9^	

Upper triangle contains pairwise coefficient of functional divergence (θ_ij_) between FFRP i and j and the standard error (SE). Lower triangle contains *p*-values for the significance that the estimate of type-1 functional divergence is greater than 0 between the two FFRPs compared.

### Genome-wide binding locations of Feast/Famine Regulatory Proteins (FFRPs)

We then mapped the genomic binding locations of all eight FFRPs from *H. salinarum* NRC-1 to understand how the homologous TFs might have diverged to perform different functions. Each FFRP was over-expressed with an epitope tag, chromatin immunoprecipitation (ChIP) was performed, and its genome-wide binding locations were mapped by tiling microarray hybridization (ChIP-chip). The over-expression of the epitope tagged FFRPs allows the identification of an FFRP’s binding sites independent of the condition in which the ChIP-chip study was performed. The genomic distribution of FFRP binding sites between intergenic and genic sequences (18% and 82%, respectively) was equivalent to the fraction of intergenic and coding sequences in the genome (14% and 86%, respectively; Additional file 5: Table S4). The eight FFRPs were found to regulate between 34 and 356 genes whose promoters harbor their experimentally mapped binding sites (i.e. when the binding site was within 250 bp upstream and 50 bp downstream of the start codon of a gene; Additional file 5: Table S4). The DNA-binding map revealed that approximately 30% of all genes (n =712) in *H. salinarum* NRC-1 were putatively regulated by one or more FFRPs. Interestingly, nearly half of these genes (i.e., 341 out of 712, permuted *p*-value <1 × 10^−5^) had at least two FFRP binding sites in their promoter region, generating a highly overlapping set of interactions. The high degree of overlap between FFRP target genes could be explained by similarity of FFRP DNA recognition motifs [23] and/or formation of hetero-oligomeric structures [13].

### Evidence of functional divergence between FFRPs

**Figure 1:**
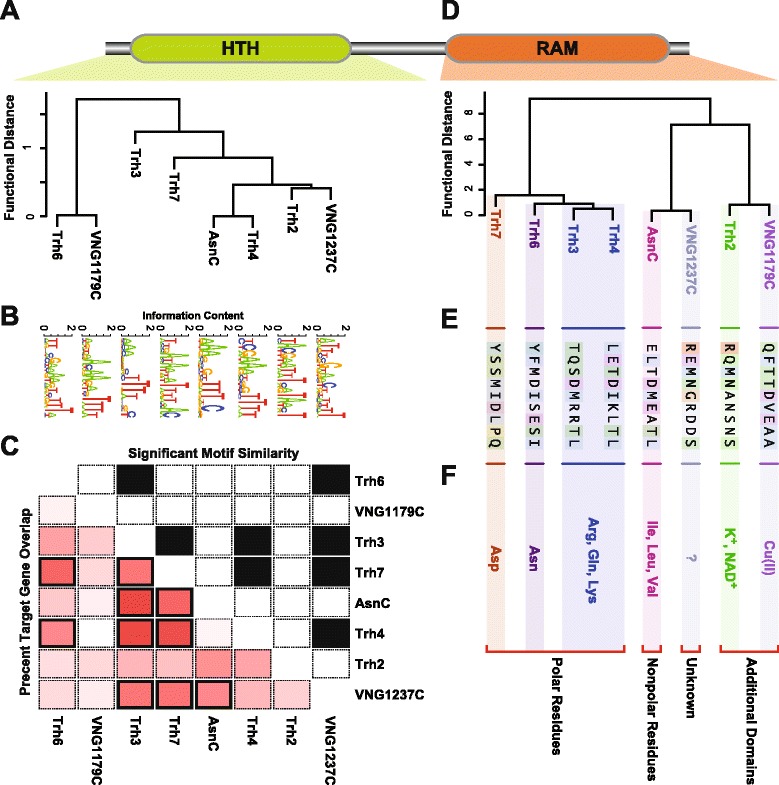
**Explaining functional and contextual divergence by clustering the functional distance of protein sequences from helix-turn-helix (HTH) DNA binding domains and RAM domains of FFRPs in** ***H. salinarum*** **. A**. Hierarchically clustered tree of the functional distance between HTH DNA binding domains of FFRPs. **B**. Putative DNA recognition motifs for each FFRP. **C**. Upper triangle of matrix displays significant pairwise FFRP DNA recognition motif similarity as black boxes (Benjamini-Hochberg corrected *p*-value ≤0.05). Lower triangle displays pairwise FFRP target gene overlap as percent overlap as intensity of red boxes and significant overlap using black outline for boxes (*p*-value ≤0.05 and percent overlap ≥50%). **D**. Hierarchically clustered tree of the functional distance between RAM domains of FFRPs. **E**. Key amino acid residues used to predict the most likely effector molecules for each FFRP. **F**. Predicted effector molecule preferences for RAM or additional domain. Arg = arginine, Gln = glutamine, Lys = lysine, Ile = isoleucine, Leu = leucine, Val = valine, Asn = asparagine, and Asp = aspartic acid. The two additional domains are predicted to sense the effector molecules: K^+^ = potassium ion, NAD^+^ = nicotinamide adenine dinucleotide, Cu(II) = copper, and ? = unkown. Interesting groupings from clustering the functional distance are denoted by a red bracket below the effector molecule preferences.

**Figure 2:**
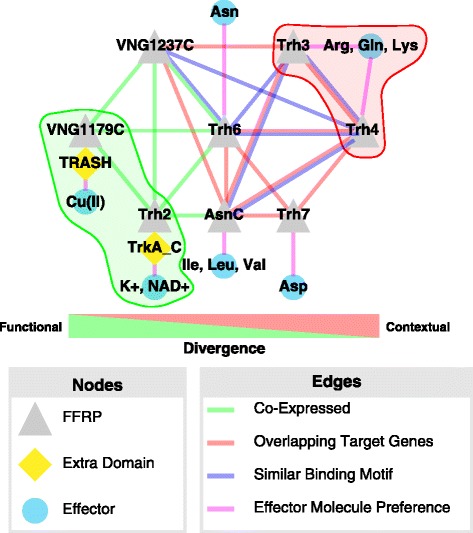
**FFRP target gene overlap and co-expression demonstrate both functional and contextual divergence in the evolution of the 8 homologous FFRPs.** Nodes are FFRPs (grey triangles), extra domains for sensing effector molecules (yellow diamonds), or effector molecules (cyan circles). Purple edges indicate the preference of an FFRP for a particular effector molecule. Interaction of an FFRP with effector molecules happens through the RAM domain or extra domains (TRASH or TrkA_C) that have fused to the FFRP. Green edges indicate significant co-expression (correlation coefficient ≥0.5 and *p*-value ≤0.05) of two FFRPs across a panel of 35 experimental contexts. Red edges indicate significant overlap (Bonferroni corrected *p*-value ≤0.05 and percent overlap ≥50%) between the experimentally determined target genes for two FFRPs. Blue edges indicate significant similarity (Bonferroni corrected *p*-value ≤0.05) between the DNA recognition motifs for two FFRPs. Below network is a scale showing that co-expression suggests functional divergence and similarity in targets and DNA recognition motifs suggests contextual divergence. Highlighted in red with a dashed red boundary is an excellent example of contextual divergence where Trh3 and Trh4 have similar target genes, DNA recognition and effector molecule preferences motifs but anti-correlated expression profiles. Highlighted in green with a dashed green border is an excellent example of functional divergence between Trh2 and VNG1179C which have similar expression profiles but different target genes, DNA recognition motifs and effector molecule preferences by fusing different extra domains.

The DNA binding domain (DBD) of the FFRP protein is a key factor in selecting the genes they modulate. We performed pairwise sequence analysis and detected significant evidence for functional divergence of the DBDs of many FFRPs (Additional file 6: Table S5). We converted the functional divergence measure into a distance metric, which we subsequently used to cluster and discover how the FFRP DBDs are related to each other (Figure 1A). Despite the functional divergence in DBD, there was significant pairwise similarity in the promoters bound by six of the eight FFRPs (AsnC, Trh3, Trh4, Trh6, Trh7, and VNG1237C; Figure 1C; Figure 2 red edges; Additional file 7: Table S6). Significant similarity in FFRP binding sites has also been observed between LrpB and LysM in *S. solfataricus* [24]. It is important to note that even with the significant pairwise similarity in promoter binding there were pairwise differences in promoter binding on the order of 36 to 100% between all FFRPs. The known ability of FFRPs to hetero-oligomerize is one possible explanation for the significant similarity in their DNA-binding locations [25]. We also investigated whether these similarities and differences across DNA-binding maps of the FFRPs could be explained by a corresponding similarity or variation in their DNA recognition motifs. The putative FFRP DNA recognition motifs (Figure 1B; Additional file 2: Figure S3) were remarkably similar to the degenerate A/T-rich core motifs that have been characterized for other FFRPs (FL10 and FL11 from *P. horikoshii* OT3, LrpB for *S. solfataricus,* and FL3 from *T. volcanium*) [23]. The motifs determined by analysis of genome-wide binding locations of the *H. salinarum* NRC-1 FFRPs also contained a highly conserved and functionally important CG present in the motifs of LrpB and LysM from *S. solfataricus* [16,26]. Notably, DNA recognition motifs of three FFRPs (Trh2, Trh6 and Trh7) were significantly similar (*p*-value ≤0.05) to characterized binding motifs for at least one of the FFRP orthologs (Additional file 8: Table S7) [23]. We observed significant pair-wise similarities between DNA recognition motifs for five FFRPs (AsnC, Trh3, Trh4, Trh6 and VNG1237C; Bonferroni corrected *p*-value ≤0.05; Figure 1C; Figure 2 blue edges; Additional file 9: Table S8). Interestingly, Trh2 and VNG1179C which have additional functional domains do not show significant overlap of target genes with other FFRPs nor do they have similar DNA recognition motifs. This could be evidence that the additional domains interfere with RAM domain mediated hetero-oligomerization which alters their function. Notwithstanding the overall similarity, subtle variations in the consensus recognition sequence motifs seem to be important as they extended regulation by each FFRP to additional unique sets of genes. For instance, consistent with functions regulated by FFRPs in other organisms [27], AsnC, VNG1237C and Trh3 were all implicated in regulation of genes with translation-associated functions, but only VNG1237C was also implicated in regulation of ‘ATP synthesis coupled proton transport’ (Additional file 10: Table S9). Thus, our data demonstrate functional divergence through subtle variations that have resulted in at least three DNA recognition motifs for the eight homologous FFRPs.

### Evidence of contextual divergence of FFRPs

One plausible explanation for the evolutionary retention of two or more FFRPs with similar target genes is that they might contribute to fitness in different contexts or respond to different effector molecules. We looked for evidence of contextual divergence by comparing the expression patterns and putative effector molecule dependencies of the eight FFRPs.

First, we computed pairwise expression correlations of the eight FFRPs across a compendium of 466 transcriptome profiles of *H. salinarum* NRC-1 from 35 different growth conditions (high temperature, copper, high H_2_O_2_, etc.; Additional file 11: Table S10) [20,21,28-36]. Interestingly, five FFRPs (AsnC, Trh2, Trh6, VNG1179C and VNG1237C) that putatively regulate different sets of genes had similar expression patterns (pair-wise correlation coefficient >0.5 and *p*-value ≤0.05; Figure 2 green edges; Additional file 2: Figure S4; Additional file 12: Table S11). By contrast, with the exception of Trh2 and VNG1237C, the expression patterns of FFRPs that regulate similar sets of genes (e.g., AsnC, Trh3 and Trh4) were not correlated (Figure 2).

Second, we observed significant evidence for functional divergence between the RAM domains of many FFRPs (Additional file 13: Table S12). Again, we converted this functional divergence measure into a distance metric and used it to analyze relationships of the RAM domains of the 8 FFRPs (Figure 1D). This led to targeted analysis of key residues in the RAM domain [13,14], which further enabled the discovery of the most likely effector molecules for each of the five FFRPs (AsnC, Trh3, Trh4, Trh6 and Trh7; Figure 1E and F; Figure 2 purple edges; Additional file 2: Figure S2). The additional TrkA-C domain [19] in Trh2 and the TRASH domain [20,21] in VNG1179C suggested that these FFRPs might regulate genes in response to changes in K+/NAD+ and metal ions (e.g. Cu(II) [20]), respectively (Figure 1F; Figure 2 purple edges; Additional file 2: Figure S2). Impressively, the structure of functional distance between FFRP RAM domains parses the FFRPs into clusters that explain their effector molecule preferences (Figure 1F). Firstly, functional distance grouped together Trh3 and Trh4 and predicted that they have similar preferences for Arg, Gln, and Lys. Similarly, Trh3, Trh4, Trh6 and Trh7 were predicted to share a preference for polar amino acids. By contrast, AsnC and VNG1237C are most likely modulated by nonpolar amino acids. Finally, the co-clustering of Trh2 and VNG1179C is most likely because they are most diverged and their RAM domains are likely non-functional. Instead, their effector molecule preferences originate from the fused domains (K+ (TrkA-C domain) for Trh2, and Cu2+ (TRASH domain) for VNG1179C). Thus, the responsiveness to different effector molecules explains how FFRPs that regulate a similar set of genes or have similar expression patterns across 35 environmental contexts (e.g. Trh6 and AsnC) might have sub- or neo-contextualized (Figure 2).

### FFRPs evolved into distinct roles through both functional and contextual divergence

Altogether, the evidence for functional and contextual divergence demonstrates that no two FFRPs are similar in all respects (Figure 2). VNG1179C and Trh2 provide an excellent example of functional and contextual divergence. The two FFRPs are highly co-expressed (correlation coefficient =0.85, *p*-value =6.7 × 10^−8^; Figure 2 red highlight with red dashed outline) but have functionally diverged because of variations in their binding motifs (*p*-value =0.68), and their functions are further contextualized by their differential responsiveness to K+ (Trh2) and Cu(II) (VNG1179C). On the other hand, Trh3 and Trh4 (Figure 2 green highlight with green dashed outline) have very similar DNA recognition motifs, similar preference for effector molecules (lysine and arginine), but have contextually diverged through differential expression across environments (correlation coefficient = -0.32, p-value =5.8 × 10^−2^). Thus, the eight FFRPs in *H. salinarum* NRC-1 have evolved to take on distinct roles based on who they regulate (variations in DNA-binding domain), when they are expressed (promoter variations), or which effector molecules modulate their activity (variations in RAM domain, or fusion of an additional effector molecule binding domain).

### Context dependent regulation of FFRP target genes

**Figure 3:**
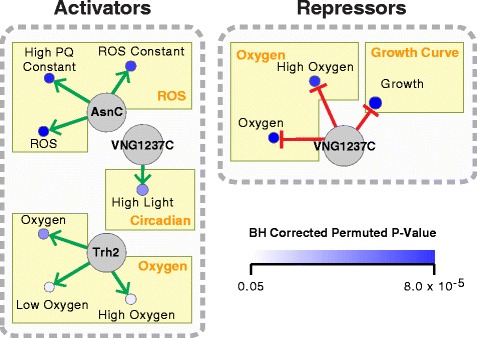
**Discovery of conditional regulation by FFRPs.** Large grey nodes represent different FFRPs, smaller blue nodes represent the contexts where the FFRPs activate (green arrow) or repress (red bars) their ChIP-chip target genes. The intensity of the blue for a context describes the significance of the regulatory interaction prediction as the Benjamin-Hochberg corrected permuted *p*-value. Context dependent regulation was identified for three of the eight FFRPs. BH: Benjamin-Hochberg, PQ: paraquat, ROS: Reactive Oxygen Species.

While we expected that over-expression of an FFRP would reveal the most comprehensive set of binding sites, we also expected that only a subset of these binding sites would be conditionally functional in any given environment. We predicted that the context in which expression of an FFRP is significantly correlated to subsets of its target genes would provide the means to identify conditionally functional binding-sites of each FFRP. We investigated patterns of correlations between each FFRP and its target genes across 35 environmental contexts, described above. We restricted our analyses to only those conditions in which expression level of the FFRP changed appreciably (1.75-fold change; Additional file 14: Table S13). Because FFRPs can function as activators [37] or repressors [38] we tested for both positive and negative correlation between expression changes of an FFRP and its target genes. Three of the eight FFRPs were significantly correlated or anti-correlated to subsets of their target genes across diverse environmental contexts (Benjamini-Hochberg corrected permuted *p*-value ≤0.05 and correlation coefficient ≤ ±0.4, Figure 3). Based on this analysis, three FFRPs (AsnC, Trh2, and VNG1237C) were predicted to function as conditional activators (Additional file 15: Table S14), while VNG1237C appears to also function as a conditional repressor for a different set of conditions (Additional file 16: Table S15). This analysis also revealed specific experimental design parameters (growth condition, phenotype, etc.) to further characterize the predicted functions of FFRPs.

### Conditional activation of 158 genes by AsnC contributes to fitness in sub-inhibitory levels of paraquat

**Figure 4:**
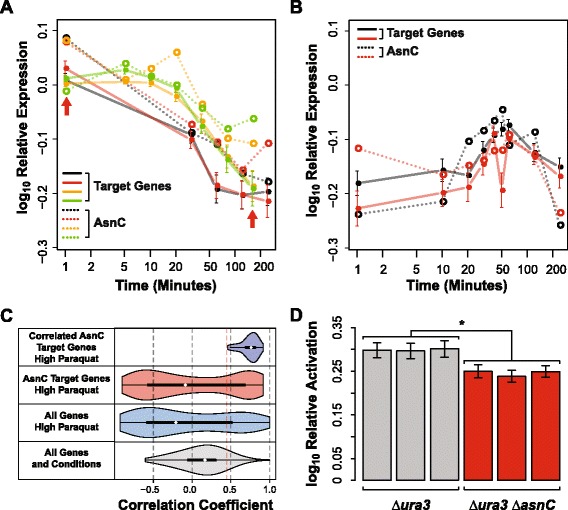
**AsnC acts as an activator of 158 genes under normal conditions and its regulation is turned off during PQ induced stress. A**. Expression of AsnC (dashed lines) and the median expression of its 158 target genes (solid lines) for four biological replicates (black, red, orange and green) are strongly correlated when 4 mM PQ is added to the culture (PQ is added at 0 minutes and first microarray sampling is -1 minutes) [35]. Red arrows indicate the sampling times of 1 and 160 minutes used for microarray validations. **B**. Expression of AsnC (dashed lines) and the median expression of its 158 target genes (solid lines) are strongly correlated when 4 mM PQ is removed and the cells are allowed to recover for 4 hours (PQ is removed at 0 minutes and first microarray sampling is taken at 0 minutes) [35]. **C**. Distribution of correlation coefficients for all 2,400 genes on the bottom (grey), all 2,400 genes in the high PQ conditions (blue), 426 AsnC target genes in the high PQ conditions (red) and the 158 correlated AsnC target genes in the high PQ conditions (purple). Red dashed line indicates median correlation coefficient of 426 AsnC target genes in the high PQ conditions used as a cutoff to discover 158 correlated AsnC target genes. **D**. Relative activation is significantly lower for the 158 AsnC target genes between *∆ura3 ∆asnC* and *∆ura3* control strain (*p*-value =0.05). ‘*’ means Wilcoxon rank-sum test *p*-value ≤0.05.

**Figure 5:**
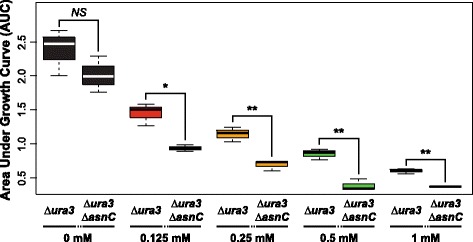
**Deletion of AsnC leads to growth defects in sub-inhibitory levels of PQ.** Growth curves were conducted for 0, 0.125, 0.25, 0.5 and 1 mM PQ and significant differences of the area under the growth curve (AUC) were observed between *∆ura3 ∆asnC* and *∆ura3* control strain only when PQ was added (*p*-value =2.2 × 10^−1^, 2.4 × 10^−2^, 5.6 × 10^−3^, 2.3 × 10^−3^, and 5.0 × 10^−3^; for 0, 0.125, 0.25, 0.5 and 1 mM PQ, respectively). ‘NS’: not significant, ‘*’: Student’s *T*-test *p*-value ≤0.05, ‘**’: Student’s *T*-test *p*-value <0.01.

Transcript levels of both AsnC and 158 of its 356 target genes decreased in response to a sub-lethal dose of the reactive oxygen species generating agent paraquat (PQ; Figure 4A) and were restored upon removal of PQ (Figure 4B) [35]. Our prediction was that differential regulation of these 158 genes by AsnC was important for oxidative stress management upon exposure to PQ (positive correlation coefficient =0.71 and Benjamini-Hochberg corrected *p*-value =6.7 × 10^−3^) (Figure 3 Activators, Figure 4). We tested this hypothesis by monitoring transcript level changes of the 158 genes in the *∆asnC* strain at 1 and 160 minutes post-addition of 4 mM PQ (red arrows in Figure 4A). We observed significant reduction (*p*-value =0.05) in activation of the 158 genes in the *∆ura3 ∆asnC* strain at 1 minute relative to 160 minutes post-addition of 4 mM PQ, validating that AsnC activates these genes early and addition of PQ turns this activation off (Figure 4D). Impressively, *∆ura3 ∆asnC* also had a PQ-dependent growth-defect (*p*-value ≤0.05, Figure 5), demonstrating the physiological importance of this conditional regulation of specific genes by AsnC in the presence of PQ.

### Conditional repression of 47 genes by VNG1237C is important for normal growth

**Figure 6:**
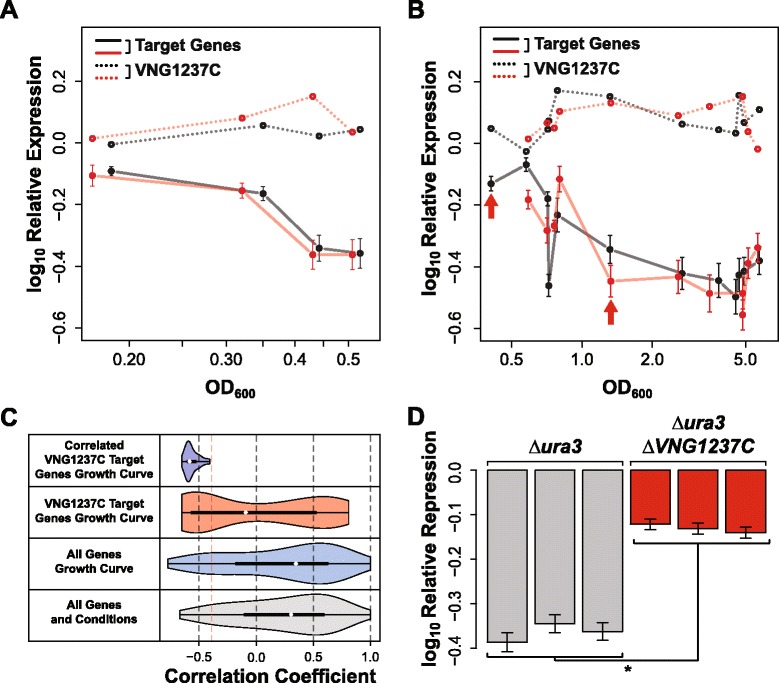
**VNG1237C acts as a repressor of 47 genes during growth in batch culture. A**. and **B**. Expression of VNG1237C (dashed lines) and the median expression of its 45 target genes (solid lines) for two biological replicates (black and red) are strongly anti-correlated across the growth curve as measured through optical density at 600 nm (OD_600_) [30,34]. Red arrows indicate the sampling OD_600_s of ~0.18 and ~1.15 used for microarray validations. **C**. Distribution of correlation coefficients for all 2,400 genes on the bottom (grey), all 2,400 genes in growth curve conditions (blue), 116 VNG1237C target genes in the growth curve conditions (red) and the 47 anti-correlated VNG1237C target genes in the growth curve conditions (purple). Red dashed line indicates median correlation coefficient of 116 VNG1237C target genes in the growth curve conditions used as a cutoff to discover 47 correlated VNG1237C target genes. **D**. Relative repression is significantly higher for the 47 VNG1237C target genes between *∆ura3 ∆VNG1237C* and *∆ura3* control strain (*p*-value =0.05). ‘*’ means Wilcoxon rank-sum test *p*-value ≤0.05.

**Figure 7:**
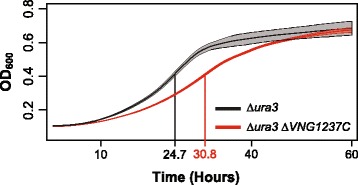
**Deletion of VNG1237C decelerates growth and delays time to maximum growth rate.** Growth rate of *∆ura3 ∆VNG1237C* was significantly decelerated (Student’s *T*-test *p*-Value =7.8 x 10^−3^) and had a significant delay of 6.1 hours in the time to maximum growth rate (Student’s *T*-test *p*-Value =8.9 x 10^−4^) in comparison to the *∆ura3* control strain. The time to maximum growth rate are shown using vertical lines to the growth curves (*∆ura3 ∆VNG1237C* strain =30.8 hours and *∆ura3* control strain =24.7 hours). Standard errors for the three biological replicates at each 30 minute time point in the growth curves are shown as shaded black or red regions.

We also performed experiments to test the predicted role of VNG1237C in repressing 47 out of its 116 target genes as cell density increased during growth in batch culture [30,34] (negative correlation coefficient = -0.57 and Benjamini-Hochberg corrected *p*-value =8.0 × 10^−5^ across 108 microarrays capturing transcriptional changes during growth; Figure 3 Repressors; Figure 6). Consistent with this prediction we observed that deletion of VNG1237C resulted in a significant loss of repression (*p*-value =0.05) of the 47 genes during growth (Figure 6D). Interestingly, the poor growth characteristics of the *∆ura3 ∆VNG1237C* strain suggested that this conditional regulation of 47 genes by VNG1237C has physiological relevance (Figure 7). We performed additional control experiments with all FFRP deletion strains to rule out that this might be a general growth defect for all FFRP deletion strains. These experiments confirmed that the growth defect was specific to the deletion of VNG1237C (combined Bonferroni corrected *p*-value ≤0.05) (Table 2).

**Table 2:** **Significance of effects of deleting VNG1237C on growth curve are not observed for other FFRPs**

**FFRP deletion strain**	**Max growth rate** ***p*** **-value**	**Time to max growth rate** ***p*** **-value**	**Area under growth curve (AUC)** ***p*** **-value**	**Combined** ***p*** **-value**
*∆asnC*	2.7 × 10^−1^	1	4.3 × 10^−2^	8.9 × 10^−2^
*∆trh2*	9.1 × 10^−1^	1	1	7.8 × 10^−1^
*∆trh3*	1	1	1	1
*∆trh4*	4.3 × 10^−2^	5.9 × 10^−1^	1.9 × 10^−1^	8.6 × 10^−2^
*∆trh6*	1	9.4 × 10^−1^	3.4 × 10^−1^	8.9 × 10^−1^
*∆trh7*	1	1.4 × 10^−2^	3.4 × 10^−1^	1.8 × 10^−1^
*∆VNG1179C*	1	1	1	1
*∆VNG1237C*	1.5 × 10^−2^	4.5 × 10^−2^	3.5 × 10^−3^	7.5 × 10^−5^

*P*-values were computed using unpaired Student’s *T*-test and Bonferroni corrected. Combined *p*-values computed using Stouffer’s Z-score method.

## Conclusions

Our results demonstrate how divergence across regulators of the FFRP family has rewired the *H. salinarum* NRC-1 gene regulatory network to differentially regulate genes and bring physiologically relevant functions to specific environmental conditions. The specialization of each FFRP function is a product of changes to three properties: 1) variations in the DNA-binding domain, which determine which promoters it binds; 2) promoter variations, which determine when it is expressed; and 3) variations in the RAM domain or fusion to an entirely different ligand-binding domain, which alters specificity for effector-molecules to determine when it is post-translationally activated or inactivated. We demonstrated significant functional divergence was present at the protein sequence level between each FFRP. Thus it is not surprising that none of the FFRPs are alike in all three respects, because variations in one or more of these three key properties has potentially given each a unique and physiologically relevant capability, such PQ-responsive regulation by AsnC and growth-specific regulation by VNG1237C. There was significant overlap between the set of genes that were conditionally regulated by AsnC and VNG1237C (p-value =2.0 × 10^−33^). In addition to regulating a common set of core functions such as ribosomes, translation factors, ATP synthase, and cobalamin biosynthesis, the two FFRPs also regulated few unique genes, such as DNA repair enzymes (AsnC) and a Na+/H + antiporter complex (VNG1237C). Yet, conditional regulation of similar genes by the two FFRPs had distinct environment-specific consequences, demonstrating how contextual and functional divergence of FFRPs has physiologically important consequences for fitness of *H. salinarum* NRC-1 under routine and stressful conditions.

Early work on the mechanisms by which TFs diverge into unique roles focused primarily on variation at the level of DNA recognition motif, i.e. functional divergence [39]. More recent work has shown that variation in the promoters of TFs altering the contexts in which they are expressed is also important, i.e. contextual divergence [2,3]. Our work demonstrates that contextual divergence can also arise from variation in ligand binding domains that result in preference for different effector molecules. Interestingly, variations in these three properties were interrelated in that divergence in one excludes the need for divergence in the other two but they are not directly predictive of each other. As was observed previously for human TF homologs [2], there is no correlation between pairwise similarity of expression and similarity of DNA recognition motifs across all FFRPs (Kendall’s tau =0.02, *p*-value =0.87). By integrating across the different levels of divergence it was possible to come up with specific predictions about physiologically relevant conditional regulation of FFRP target genes. For instance, AsnC was down-regulated in response to PQ treatment, and the down-regulation of its target genes in this context ultimately influenced the fitness of *H. salinarum* under oxidative stress conditions. Similarly, VNG1237C was up-regulated during growth, it was active during growth, and the repression of its target genes was important for wild type growth characteristics. These interrelationships underlie the success and power of our strategy of integrating binding and gene expression data to elucidate conditional regulation by each member of this expanded FFRP family of regulators. Specifically, by over-expressing a tagged TF we were able to generate comprehensive DNA-binding maps in a relatively context independent manner. The assessment of correlation between transcriptional changes of the FFRP and its target genes revealed the subset of binding events that were conditionally functional in specific environmental contexts. This is a generic strategy that can readily be applied to elucidate conditionally active gene regulatory networks in any organism, just from a map of genome-wide TF-DNA binding locations and a compendium of transcriptome profiles from diverse environments. Future studies employing ChIP-exo [40] will enhance this generic strategy by improving the resolution of FFRP binding events which makes the discovery of FFRP target genes and motifs more straightforward.

While we had some success in identifying the effector molecules for each FFRP homolog, these predictions will have to be experimentally verified [6,9-18]. Recent studies in a closely related species *H. salinarum* R1 validated the preference of Trh4 for glutamine and determined that the novel effector molecule binding residues of Trh7 indicate a preference for aspartic acid [14]. The preferences, specificity, and affinity for effector molecules, and how they influence the activity of an FFRP are critical pieces of information as they will shed insight into how an organism senses and responds to dynamic intra-cellular and external environmental changes. The systems level integration and coordination of varied functions of expanded FFRPs, as well as of all other regulators will ultimately reveal how variations in the promoter, DNA-binding domain and effector-molecule recognition in an FFRP co-evolve to generate novel coordination of different cellular processes that is better suited for the new environment.

## Methods

### Evolutionary analyses of FFRPs in *H. salinarum* NRC-1

Protein sequences were collected from MicrobesOnline [41] for FFRPs and closely related homologs from other species. Homologs from other species were discovered using FastBLAST [41] with ≥50% identity with an FFRP in H. salinarum NRC-1. Using MEGA6 [42] protein sequences were aligned using MUSCLE [43] and a phylogenetic tree was reconstructed using the Minimum Evolution method [44]. The aligned protein sequences and phylogenetic tree were then used to compute type-I functional divergence coefficients (θ_I_) using the maximum likelihood based method in the DIVERGE [22] software package. Significance of type-I functional divergence coefficients were calculated using a chi-square test from the log of the likelihood ratio test statistic [22]. Pairwise functional distances were calculated from type-I functional divergence as the –ln(1 – θ_ij_) [22], where i and j are FFRPs. Functional distances were then converted to a distance matrix and clustered using complete linkage hierarchical clustering. Analyses were repeated for the DBD and RAM domain by restricting analyses to only those protein coding sequences corresponding to those regions as described on MicrobesOnline [41].

### Strains construction and culturing conditions

The two FFRP deletion strains for *asnC* and *VNG1237C* were derived from *H. salinarum* NRC-1 *∆ura3* strain via a two-step gene replacement strategy [20] using the primers and plasmids described in (Additional file 17: Table S16). C-Myc tagged strains for ChIP-chip were constructed as previously described [34] using the primers and plasmids described in Additional file 18: Table S17. All *H. salinarum* NRC-1 strains were cultured in standard growth conditions in complete medium (CM, 250 NaCl, 20 g/L MgSO_4_ 7H2O, 3 g/L sodium citrate, 2 g/L KCl, 10 g/L peptone) or complete defined medium with 19 amino acids (CDM, Additional file 19: Table S18). All deletion strains and the control/parental strains *(∆ura3*) of deletions were grown in CM or CDM with an added 0.05 mg/ml uracil to compensate for their uracil deficiency.

### Genome wide FFRP binding site analysis (ChIP-chip)

Chromatin immuno-precipitation and microarray hybridization (ChIP-chip) experiments were carried out for all 8 FFRPs in *H. salinarum* NRC-1 using the Agilent-030521 Halobacterium sp. NRC-1 Tiling V1 013324 array (GPL13426) [33]. To study the FFRP localization in both nutrient replete and deplete conditions, all strains were grown in CDM, and samples were harvested in both early log phase and late log phase. ChIP of c-Myc tagged protein complexes were conducted as described [33,45]. Enriched DNA from ChIP complexes and unenriched non-IP DNA were each labeled and hybridized to the whole genome tiling array (GSE62052). Each ChIP-chip consisted of at least two independent biological replicates, with at least 16 replicate spots in each. Resulting localization data was median normalized and further analyzed for statistically significant enrichment using MeDiChI, a regression model that learns a generative model of joint binding events [46]. MeDiChI provides a list of putative binding sites with a resolution of 50 bp. By calculating the average intergenic region upstream of the transcriptional start site for all genes in *H. salinarum* NRC-1 the promoter region was determined to be ~200 bp (195 bp) and added a 50 bp buffer to each end accounting for the resolution of MeDiChI for a total or –250 bp upstream and +50 bp downstream of the transcriptional start site. Genes targeted by a particular FFRP were identified as having a MeDiChI *p*-value ≤0.01 in their promoter region or by being part of an operon with a binding site in the promoter region of an upstream gene in the operon. Pairwise FFRP overlap was computed using hypergeometric enrichment *p*-values and percent overlap uses the smallest target gene set as the total.

### Functional enrichment analysis

We used the Bioconductor package topGO [47] to discover significantly enriched GO biological process terms in gene sets of interest.

### Discovering FFRP DNA recognition motifs from ChIP-chip target genes

Discovery of putative DNA recognition motifs was conducted as described in Ashworth, et al. 2014 [48]. Briefly, gene promoters in Halobacterium salinarum were considered binding targets if a ChIP-chip peak with a *p*-value less than 0.10 was present within 100 bp upstream of the transcriptional start site. For de novo discovery of genome-wide promoter DNA binding sites from ChIP experiments for each FFRP, MEME [49] was performed on the upstream non-coding promoter sequences of all genes with evidence of ChIP binding. The following parameters were used to run MEME: -minw 13, -maxw 17, -nmotifs 2, and MEME was supplied with a first-order background Markov model computed over all input sequences. Upstream sequence regions tested for de novo motif detection were –100 to 0 bp relative to gene CDS starts or transcriptional start sites. For the purpose of inferring gene promoters which are bound by FFRPs, FIMO was used [49] to identify potential FFRP transcription factor binding sites (TFBS) in promoter regions from DNA-binding position weight matrices (PMWs) with a motif p-value below the default threshold (1×10^−4^). The similarity between DNA recognition motifs was computed using TOMTOM [50].

### Discovering contextual divergence across FFRPs

Similarity in expression between FFRPs was computed though pair-wise Spearman’s rank based correlations of expression between all eight FFRPs across 35 condition sets (Additional file 11: Table S10). A correlation coefficient greater than or equal to 0.5 and p-value less than or equal to 0.05 was used as a cutoff for significant similarity between two FFRPs expression.

Discovery of putative effector molecules for FFRPs utilized previous work that discovered a set of nine key amino acids that specify the code for effector molecule preference [13]. First, all eight FFRP protein sequences were aligned with five homologous FFRPs (Additional file 2: Figure S2) [13]. Then similarity between the nine effector molecule specifying residues were scored using the BLOSSUM62 matrix and average distance hierarchical clustering was used to define clusters of FFRPs with similar effector molecule preferences.

### Discovering context dependent regulation by FFRPs

In total 35 condition sets were utilized for the analysis (Additional file 11: Table S10). These condition sets were further filtered to include only sets where the expression of a FFRP has at least 1.75 fold-change in expression across a condition set. We chose to use 1.75 fold-change as it provided a reasonable number of 127 filtered experimental contexts where an FFRP significantly changed. This resulted in 24, 26, 12, 14, 9, 4, 14, and 21 EO terms for AsnC, VNG1179C, VNG1237C, Trh2, Trh3, Trh4, Trh6, and Trh7 (Additional file 14: Table S13). Median correlation coefficient between the expression of a FFRP and its ChIP-chip targets were calculated for each filtered condition set. Positive and negative correlations were calculated separately, where a positive correlation indicates an activator role and a negative correlation co-efficient indicates a repressor role. Median correlation coefficients between a FFRP and its ChIP-chip targets in a condition set were compared to randomly sampled gene sets of the same size. In total 100,000 permutations were performed and significance of the median correlation coefficient between a FFRP and its target genes under each condition set was calculated based on the resulting permuted *p-*values (Benjamini-Hochberg multiple hypothesis correction ≤0.05). As a final filter the variance of an FFRP’s target genes were required to be significantly correlated with the FFRP’s expression (Benjamini-Hochberg multiple hypothesis correction ≤0.05). Genes whose magnitude of correlation coefficient was greater than the median correlation coefficient of the FFRP’s target genes were considered to be significantly correlated with the FFRP’s expression under that condition set.

### Microarray analysis of FFRP deletion strains in relevant condition sets

Total RNA was prepared from cell pellets using the mirVANA RNA kit (Ambion, Austin, TX) according to the manufacturer's instructions. Whole-genome tiling array, RNA labeling, hybridization and normalization were conducted as previously described [51]. All microarray conditions were collected in biological triplicates. The *asnC* deletion strain (*∆ura3 ∆asnC*) and the control *∆ura3* strain were grown in 4 mM paraquat (PQ) and were sampled at 1 and 160 minutes after PQ addition. Relative activation was calculated for each correlated ChIP-chip target gene separately as the expression level at the 1 minute time point minus the 160 minute time point. The *VNG1237C* deletion strain (*∆ura3 ∆VNG1237C*) and the control *∆ura3* strain were grown in CM media and sampled when the *∆ura3* optical density at 600 nm (OD_600_) ~0.18 and ~1.15 (corresponding *∆VNG1237C* OD_600_ ~ 0.15 and 0.69, respectively). Sampling timing for *∆VNG1237C* was designed such that the starting OD_600_s were as similar as possible and once an OD_600_ of 1.17 was reached for *∆ura3* the *∆VNG1237C* was sampled at this same time point. Relative repression was calculated for each correlated ChIP-chip target gene separately as the expression level at the larger OD_600_ minus the starting OD_600_. Significant differences between the relative activation and repression were calculated by taking the median of all correlated target genes and comparing the deletion FFRP strain to the *∆ura3* control strain using an un-paired one-sided Wilcoxon rank-sum test. Deletion microarray data reported in this paper have been deposited in the National Center for Biotechnology Information (NCBI) Gene Expression Omnibus (GEO) as GSE62052.

### Growth curve analysis

Growth assays were performed in multi-plex using a Bioscreen C instruments (Growth Curves USA, Piscataway, NJ). Each experimental condition was done in technical duplicate and biological triplicate. Cultures for inoculation were titrated from starter cultures to have a similar OD_600_ which greatly increased the consistency and reproducibility of the growth curve experiments. OD_600_ was measured every 30 minutes for 4 days after inoculation with a starting OD_600_ of 0.09. We have developed an R package entitled ‘Growth Curve Analysis Function’ that automates the extraction of the parameters maximum growth rate, time to maximum growth rate and area under the growth curve [51]. Technical duplicates were collapsed based upon the mean of the values. All three parameters were calculated for each set of biological triplicates and then the deletion FFRP strain to the *∆ura3* control strain were compared using an un-paired two-sided Student’s *T*-test.

### Availability of supporting data

Genome-wide ChIP-chip binding profiles for each FFRP studied and expression profiles for knock-out validation studies are provided under GSE62052. The 446 expression profiles from the 35 different experimental conditions are available under GSE1040, GSE4890 to GSE48900, GSE4925, GSE5557, GSE5924, GSE5925, GSE5929, GSE6776, GSE7559, GSE7609 to GSE7613, GSE7709 to GSE7740, GSE29706, GSE13150, and GSE17515.

## Additional files

Additional file 1: Table S1. Number of FFRPs for archaeal species. Additional file 2: Figure S1. Domain structures for H. salinarum NRC-1 FFRPs protein. **Figure S2.** Prediction of FFRP effector molecule(s). **Figure S3.** Similarity of discovered FFRP motifs to orthologous FFRPs. **Figure S4.** Expression of FFRPs across 35 growth conditions. Additional file 3: Table S2. Proteins from UniProt with the same domains as Trh2 (IPR000485 and PF02080). Additional file 4: Table S3. Proteins from UniProt with the same domains as VNG1179C (IPR000485 and IPR011017). Additional file 5: Table S4. FFRP ChIP-Chip target genes. Additional file 6: Table S5. Estimation of type-I functional divergence coefficent (site specific rate shift of amino acids) between the HLH DNA binding domains of 8 FFRPs from H. salinarum NRC-1. Additional file 7: Table S6. Overlap of FFRP ChIP-Chip determined target genes. Additional file 8: Table S7. Comparison of FFRP ChIP-chip derived motifs to FFRP motifs from other species determined by SELEX. P-Values are from TOMTOM, Benjamini-Hochberg corrected, and p-values ≤0.05 were considered significantly similar. pMTF is an empty vector control that demonstrates the motif similarity comes from the inserted FFRP proteins. Additional file 9: Table S8. FFRP target gene enriched DNA sequence motif similarity computed through TOMTOM P-Values. Additional file 10: Table S9. Enriched Gene Ontology Biological Processes for FFRP ChIP-chip target genes. Additional file 11: Table S10. Description of all 35 environmental contexts explored for conditional regulation. Microarray names are concatenated by semi-colons. Additional file 12: Table S11. FFRP Expression Pattern Correlation Coefficients. Additional file 13: Table S12. Estimation of type-I functional divergence coefficent (site specific rate shift of amino acids) between the RAM domains of 8 FFRPs from H. salinarum NRC-1. Additional file 14: Table S13. Environmental contexts with a significant change in FFRP expression (FC ≥ +/- 1.75). Additional file 15: Table S14. Conditional activation by an FFRP on its target genes. Additional file 16: Table S15. Conditional repression by an FFRP on its target genes. Additional file 17: Table S16. Plasmids and primers used to construct FFRP deletion strains. Additional file 18: Table S17. Plasmids and primers used to construct c-Myc over-expression for ChIP-chip studies. Additional file 19: Table S18. Complete defined media (CDM) recipe.
