# Supplementary material for: Evolution of context dependent regulation by expansion of feast/famine regulatory proteins
Source: BMC Syst Biol. 2014 Nov 14;8:122. doi: 10.1186/s12918-014-0122-2 (PMC4236453; doi:10.1186/s12918-014-0122-2)
Supplement: Additional file 2: Figure S1. — Domain structures for H. salinarum NRC-1 FFRPs protein. Figure S2. Prediction of FFRP effector molecule(s). Figure S3. Similarity of discovered FFRP motifs to orthologous FFRPs. Figure S4. Expression of FFRPs across 35 growth conditions. [file 12918_2014_122_MOESM2_ESM.docx]

**Additional Figures: Evolution of Context Dependent Regulation by Expansion of Feast/Famine Regulatory Proteins**

Christopher L Plaisier ^1a^ (cplaisier@systemsbiology.org),

Fang-Yin Lo ^1, 2a^ (fylo@systemsbiology.org),

Justin Ashworth ^1^ (justin.ashworth@systemsbiology.org),

Aaron N. Brooks ^1,2^ (abrooks@systemsbiology.org),

Karlyn D. Beer ^1,2^ (kbeer@systemsbiology.org),

Amardeep Kaur ^1^ (amardeep.kaur@systemsbiology.org),

Min Pan^1^ (min.pan@systemsbiology.org),

David J. Reiss ^1^ (david.reiss@systemsbiology.org),

Marc T. Facciotti ^3,4^ (mtfacciotti@ucdavis.edu),

Nitin S. Baliga ^1,2,5,6,*^ (nitin.baliga@systemsbiology.org)

*^1^ Institute for Systems Biology, Seattle WA; ^2^ Molecular and Cellular Biology Program, University of Washington, Seattle, WA; ^3^* Department of Biomedical Engineering and *^4^*Genome Center, *University of California, Davis, CA; ^5^Department of Microbiology, and ^6^Department of Biology, University of Washington, Seattle, WA*

*corresponding author: [nbaliga@systemsbiology.org](mailto:nbaliga@systemsbiology.org)

^a^ These authors contributed equally to this work

**Table of Contents**

**Additional Figures**

| **Page** | **Additional Figure** |
| --- | --- |
| 3 | **Figure S1.** Domain structures for H. salinarum NRC-1 FFRPs protein. |
| 4 | **Figure S2.** Prediction of FFRP effector molecule(s). |
| 5 | **Figure S3.** Similarity of discovered FFRP motifs to orthologous FFRPs. |
| 6 | **Figure S4.** Expression of FFRPs across 35 growth conditions. |

**Additional References** … Page 7

**Additional Figures**

**Figure S1.** Protein domain structure for the eight full length FFRPs in *H. salinarum* NRC-1 (AsnC, Trh2, Trh3, Trh4, Trh6, Trh7, VNG1179C and VNG1237C) and a putative FFRP homolog that is missing the DNA binding domain (Trh5). Each protein is layed out as a grey line with domains overlaid on top. Structurally FFRP proteins are comprised of a helix-turn-helix (HTH) DNA binding domain (green domain) connected through a flexible linker to a “regulation of amino acid metabolism” (RAM) domain (orange domain) that typically binds amino acids to modulate regulatory activity [1–4]. In addition Trh2 and VNG1179C have an extra TrkA-C and TRASH domain, respectively. Asterisks next to the RAM domain indicate lower similarity with the query RAM domain PF01037 from PFAM.


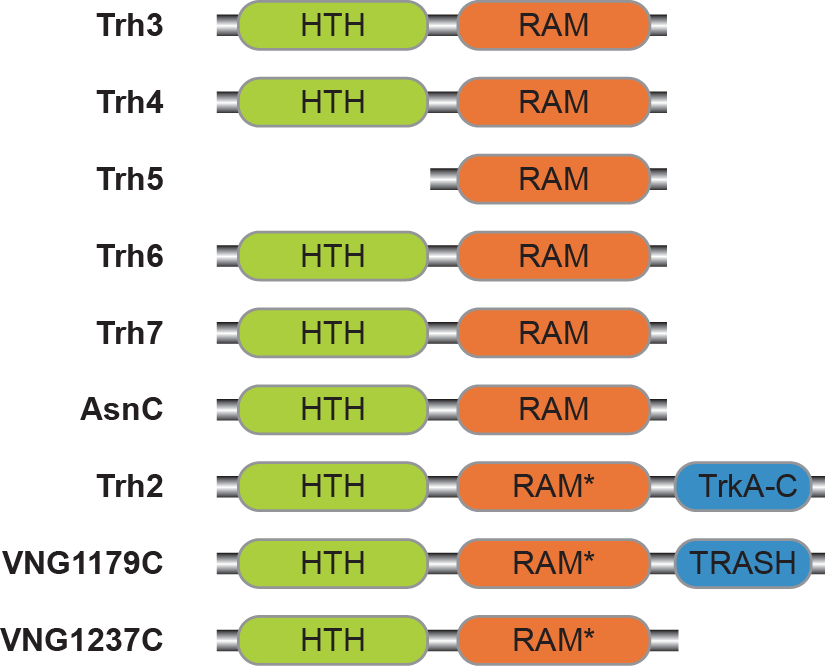


**Figure S2.** Prediction of FFRP effector molecule(s). By analyzing the key residues in the RAM domain [3, 4] of each FFRP and comparison to orthologous FFRPs we were able to assign the most likely effector molecules for five FFRPs (AsnC, Trh3, Trh4, Trh6 and Trh7). Effector molecule(s) are shown in the first column. There are eight amino acids sensed through the RAM domain: Arg = arginine, Gln = glutamine, Lys = lysine, Ile = isoleucine, Leu = leucine, Val = valine, Asn = asparagine and Asp = aspartic acid. Additionally the two extra domains are predicted to sense additional effector molecules: K^+^ = potassium ion and NAD^+^ = nicotinamide adenine dinucleotide. Using this approach it was not possible to assign an effector molecule to VNG1237C (?). Asterisk indicates that the effector molecule was assigned through an additional reference [4]. The second column is the dendrogram created by heriarchical clustering of the nine effector molecule interacting residues. The red dashed line was the threshold used to cut the dendrogram and discover similar effector molecule preferences. The third column is the FFRP names where the *H. salinarum* NRC-1 are bolded and orthologous FFRPs have their species names in parentheses. In the fourth column are effector molecule interacting residues for each FFRP.


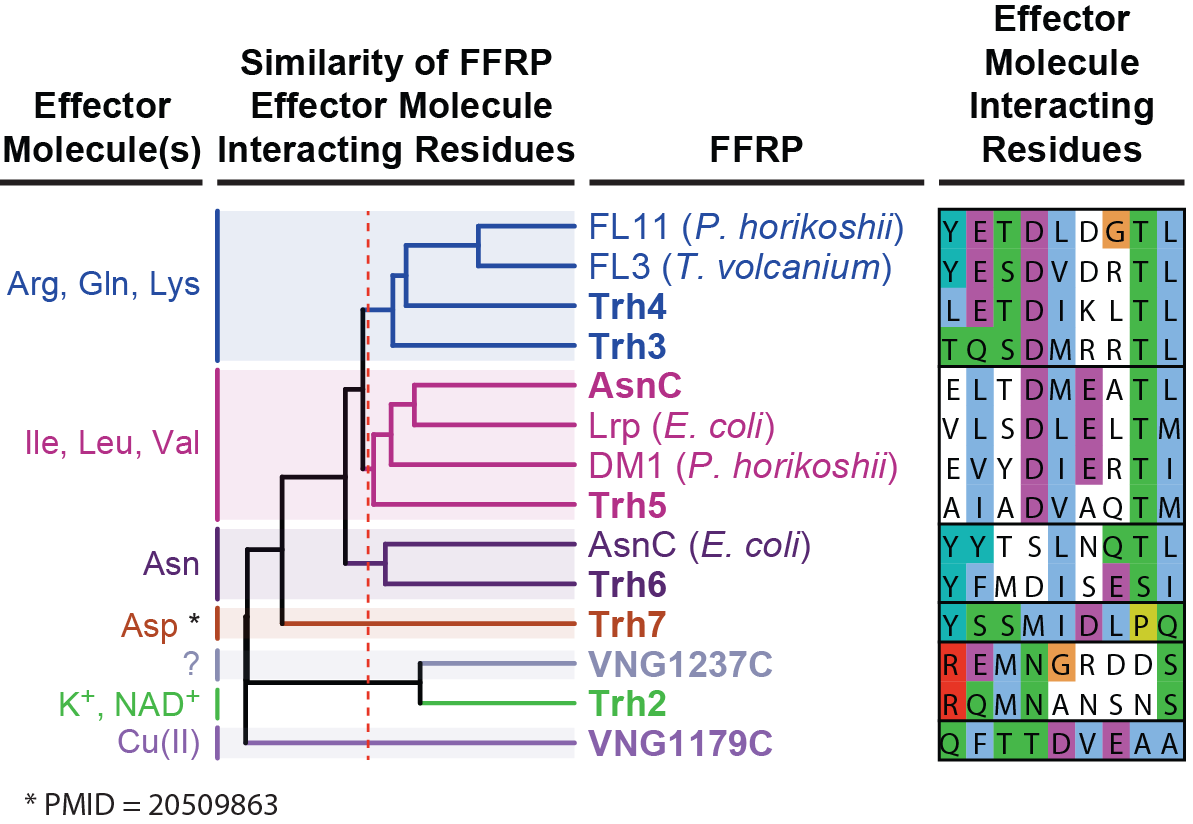


**Figure S3.** Similarity of discovered FFRP motifs to orthologous FFRPs. The motifs discovered for each *H. salinarum* NRC-1 FFRP are shown on the left (I.C. = Information Content in bits). Significant similarity with an orthologous FFRP (TOMTOM *p*-value ≤ 0.05) are shown on the right as a black box. Species abbreviations for orthologous FFRPs are described below.


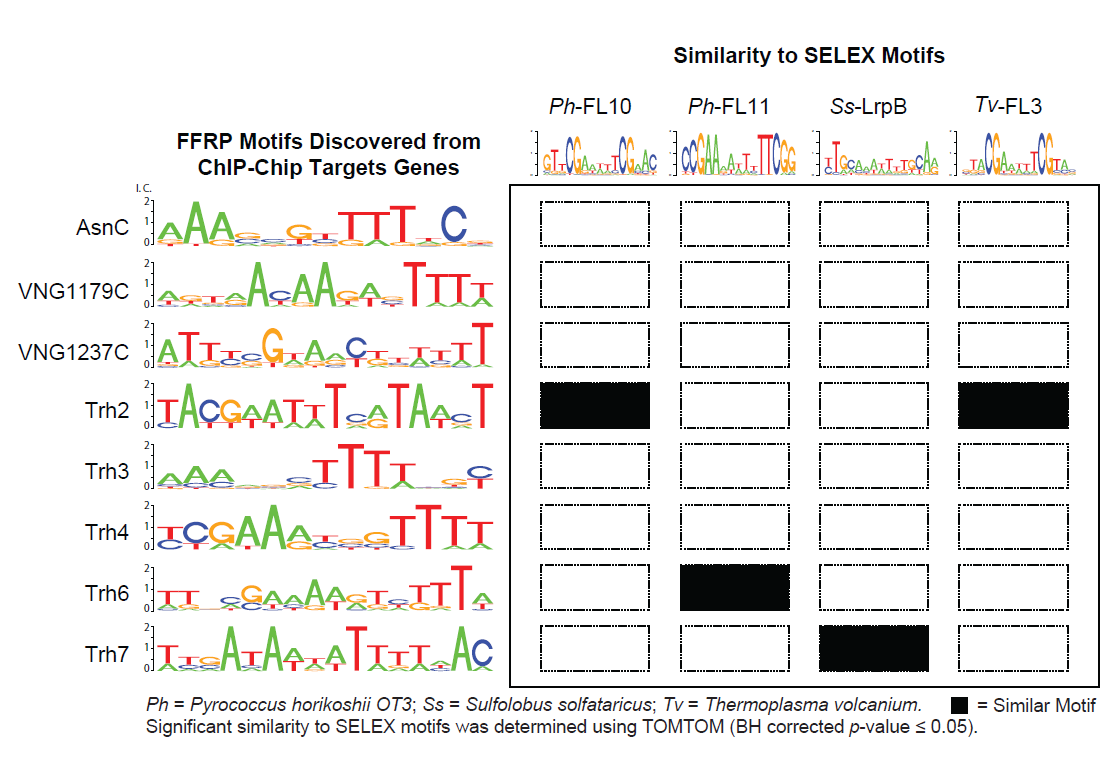


**Figure S4.** Expression of FFRPs across 35 growth conditions. Each box in the heatmap corresponds to the average expression of the FFRP, specified on the bottom, in a specific growth condition, specified on the right. Legend for relative expression is shown on the right. Dendrograms are generated from the hierarchical clustering that was used to order the FFRPs based upon similarity of expression across the 35 growth conditions.


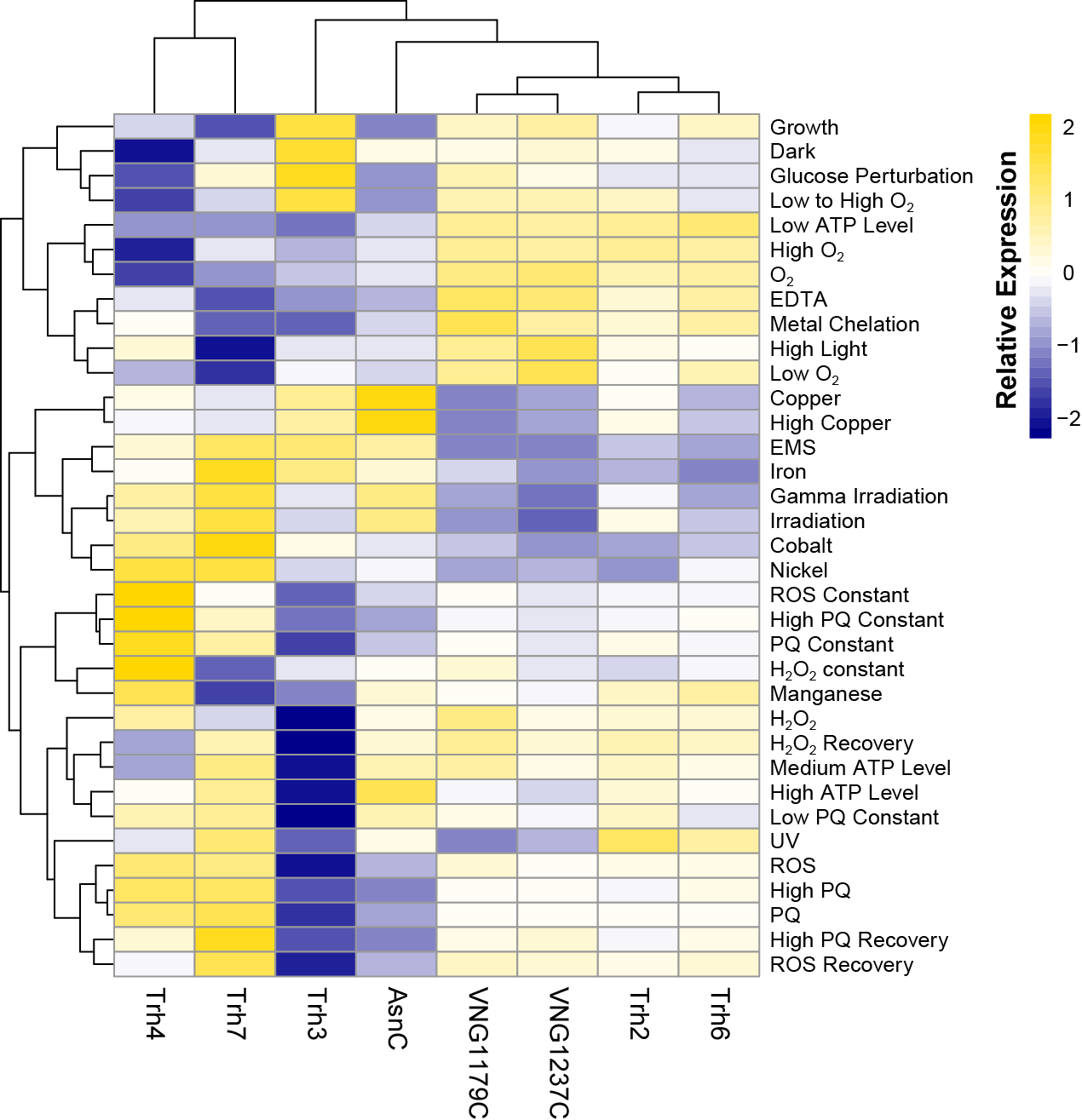


**Supplementary References**

1. Leonard PM, Smits SH, Sedelnikova SE, Brinkman AB, de Vos WM, van der Oost J, Rice DW, Rafferty JB: **Crystal structure of the Lrp-like transcriptional regulator from the archaeon *Pyrococcus furiosus***. *EMBO J* 2001, **20**:990–997.

2. Ettema TJG, Brinkman AB, Tani TH, Rafferty JB, Van Der Oost J: **A novel ligand-binding domain involved in regulation of amino acid metabolism in prokaryotes**. *J Biol Chem* 2002, **277**:37464–37468.

3. Okamura H, Yokoyama K, Koike H, Yamada M, Shimowasa A, Kabasawa M, Kawashima T, Suzuki M: **A structural code for discriminating between transcription signals revealed by the feast/famine regulatory protein DM1 in complex with ligands**. *Struct Lond Engl 1993* 2007, **15**:1325–1338.

4. Schwaiger R, Schwarz C, Furtwängler K, Tarasov V, Wende A, Oesterhelt D: **Transcriptional control by two leucine-responsive regulatory proteins in *Halobacterium salinarum* R1**. *BMC Mol Biol* 2010, **11**:40.
